# Tracking protein–protein interactions by NMR: conformational selection in human steroidogenic cytochrome P450 CYP17A1 induced by cytochrome *b*_5_†‡

**DOI:** 10.1039/d4cp01268b

**Published:** 2024-05-31

**Authors:** Alaina M. Richard, D. Fernando Estrada, Liam Flynn, Susan Sondej Pochapsky, Emily E. Scott, Thomas C. Pochapsky

**Affiliations:** a Chemical Biology Program, University of Michigan Ann Arbor MI 48109 USA; b Department of Medicinal Chemistry, University of Kansas 1251 Wescoe Hall Drive Lawrence Kansas 66045 USA; c Department of Chemistry, Brandeis University 415 South St. Waltham MA 02454-9110 USA pochapsk@brandeis.edu; d Department of Biochemistry and Rosenstiel Basic Medical Sciences Research Center, Brandeis University 415 South St. Waltham MA 02454-9110 USA; e Departments of Medicinal Chemistry, Pharmacology, and Biological Chemistry, University of Michigan Ann Arbor MI 48109 USA

## Abstract

The human steroidogenic cytochrome P450 CYP17A1 catalyzes two types of reactions in the biosynthetic pathway leading from pregnenolone to testosterone and several other steroid hormones. The first is the hydroxylation of pregnenolone or progesterone to the corresponding 17α-hydroxy steroid, followed by a lyase reaction that converts these 17α-hydroxy intermediates to the androgens dehydroepiandrosterone and androstenedione, respectively. cytochrome *b*_5_ (cyt*b*_5_) is known to act as both an effector and electron donor for the lyase oxidations, markedly stimulating the rate of the lyase reaction in its presence relative to the rate in its absence. Extensive sequential backbone ^1^H,^15^N and ^13^C nuclear magnetic resonance assignments have now been made for oxidized CYP17A1 bound to the prostate cancer drug and inhibitor abiraterone. This is the first eukaryotic P450 for which such assignments are now available. These assignments allow more complete interpretation of the structural perturbations observed upon cyt*b*_5_ addition. Possible mechanism(s) for the effector activity of cyt*b*_5_ are discussed in light of this new information.

## Abstract 2

Extensive sequential NMR resonance assignments in the human steroidogenic cytochrome P450 CYP17A1 permit detailed analysis of the interaction of cytochrome *b*_5_ with CYP17A1 that stimulates the ultimate production of testosterone and estrogen.

## Graphical abstract

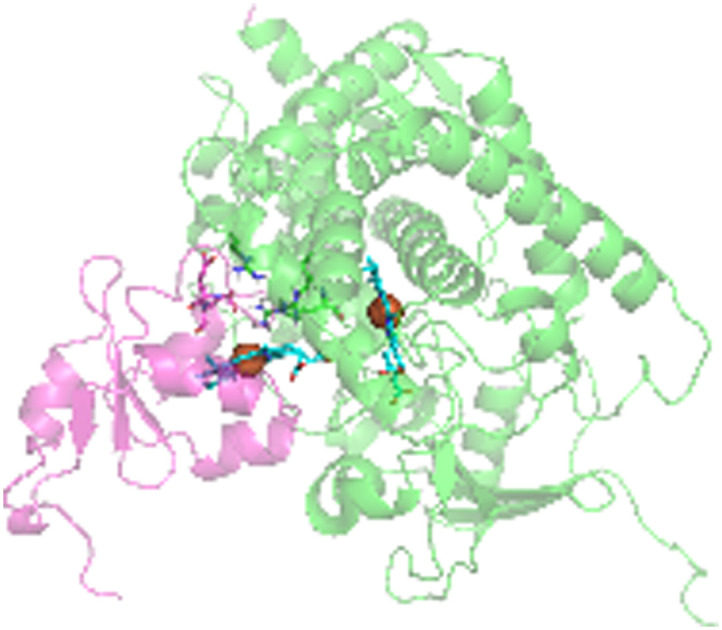

The cytochrome P450 (CYP) superfamily is notable for the vast repertoire of reactions catalyzed by these heme-containing monooxygenases. They act on a multitude of substrates, often in a highly regio- and stereospecific fashion.^1^ The chemistry catalyzed by CYPs involves the activation of molecular oxygen, a function potentially dangerous both for the enzyme and to the organism itself.

The human steroidogenic P450 CYP17A1 catalyzes two separate oxidative steps on the steroid biosynthetic pathway. The first is hydroxylation at the C17-position of pregnenolone 1̲ or progesterone 4̲ yielding the corresponding 17α-hydroxysteroids 2̲ and 5̲, respectively (Fig. 1). The second step is a lyase reaction, further oxidizing at the same 17α position, resulting in the loss of acetate and formation of dehydroepiandrosterone (DHEA) 3̲ or androstenedione 6̲. These oxidations represent a critical branch point in steroid biosynthesis. If both hydroxylation and lyase reactions occur, the physiological result is the biosynthesis of androgens including testosterone and dihydrotestosterone and subsequent production of estrogens. If the 17α-hydroxylation takes place but not the lyase reaction, then the 17α-hydroxy intermediates are converted into the mineralocorticoid cortisol. If neither oxidation occurs, the result is ultimately production of the glucocorticoid aldosterone.

**Fig. 1:**
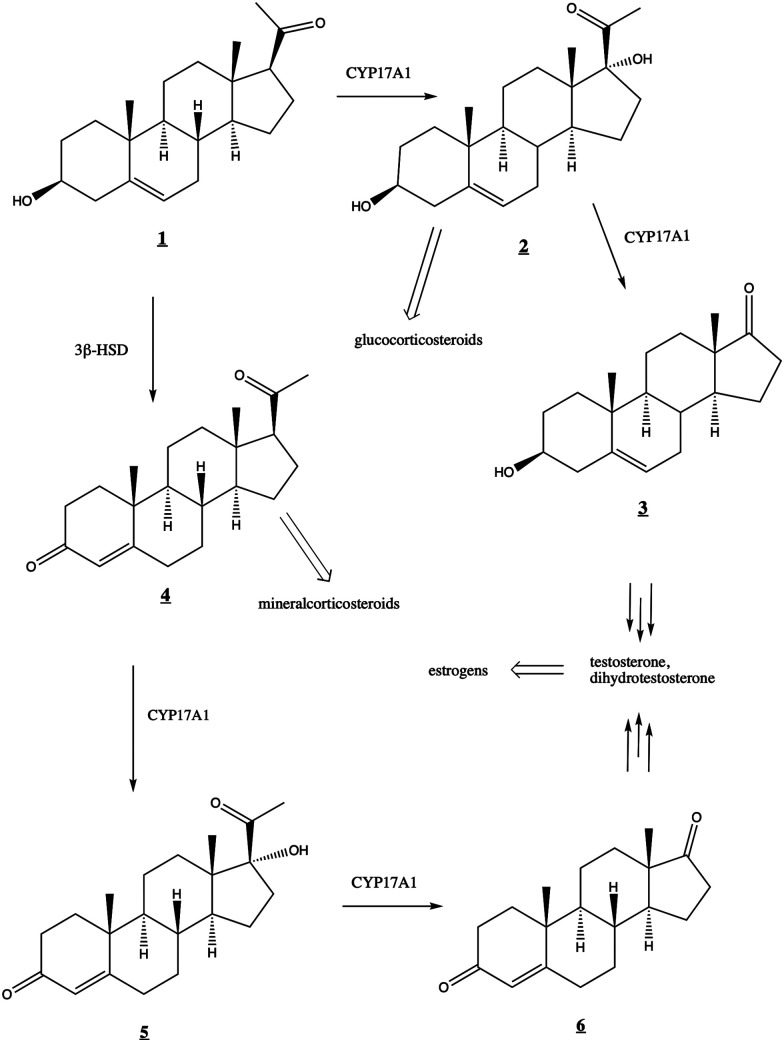
Roles of CYP17A1 in steroid biosynthetic pathway leading to testosterone, estrogens, glucocorticoids, and mineralocorticoids. 3β-HSD (3-beta-hydroxysteroid dehydrogenase) converts 1̲ to 4̲.

While both the hydroxylation and lyase reactions require NADPH-cytochrome P450 reductase (CPR) as the primary CYP17A1 redox partner, the regulatory role of the small (15 kDa) cytochrome *b*_5_ (cyt*b*_5_) is more complex. Cyt*b*_5_ enhances the rate of the lyase reaction by as much as 10- to 13-fold without significant effects on the hydroxylation reaction.^2,3^ Cyt*b*_5_ expression is strongly upregulated at adrenarche^4^ and this rate enhancement is thought to be physiologically relevant to the regulation of androgen and estrogen production.

Solution NMR has proven to be a powerful tool for characterizing structural and dynamic changes associated with the regulation of CYP function.^5,6^ A critical first step in such efforts is the comprehensive sequential assignment of backbone ^1^H–^15^N amide correlations in the enzyme. These assignments allow for the localization of perturbations as a function of such events as substrate, effector or inhibitor binding. Partial assignments for human CYP17A1 were previously determined and used to characterize several enzyme states.^7^ Herein we report on the application of multidimensional NMR methods to make additional assignments for human CYP17A1, allowing us to more completely interpret previously reported spectral perturbations resulting from the interaction of CYP17A1 with cyt*b*_5_.^7–10^

## Methods

### Preparation of selective and uniformly ^15^N labeled CYP17A1 and NMR detection of perturbations induced by cyt*b*_5_

The methodology used to prepare selective and uniformly ^15^N-labeled CYP17A1, as well as the NMR experiments employed for detecting cyt*b*_5_-induced spectral perturbations, have been described previously.^7,9^ Briefly, N-terminally truncated and C-terminally his-tagged human CYP17A1 was recombinantly expressed along with chaperones groEL and groES in either JM109 or DH5α *E. coli* using appropriately labeled defined media in the presence of the inhibitor abiraterone. This complex was purified *via* nickel affinity, cation exchange, and size exclusion chromatography. Samples of 200–300 μM CYP17A1/abiraterone were exchanged into a low-salt and glycerol-depleted buffer for improved compatibility for NMR experiments. After collecting an initial HSQC spectrum in the absence of cyt*b*_5_, the experiment was repeated after addition of 0.4 molar equivalents of cyt*b*_5_. We note that higher relative concentrations of cyt*b*_5_ led to loss of some signals being monitored due to line broadening. All ^1^H–^15^N-TROSY-HSQC spectra were collected at 40 °C on a Bruker Avance 800 MHz spectrometer equipped with a TCI cryoprobe at the University of Kansas Biomolecular NMR Core Laboratory. Raw FIDs were processed using NMRPipe^11^ and analyzed herein using TopSpin 4.1.4 (Bruker, Billerica, MA), NMRViewJ,^12^ and CcpNMR 3.1.3.^13^

### Expression and preparation of uniformly ^2^H, ^13^C, ^15^N-labeled CYP17A1

Kanamycin (kan)-resistant *E. coli* strain NCM533 was first transformed with plasmid pG-KJE8 that confers chloroamphenicol (chl) resistance and carries the tetracycline (tet)-inducible *groES* and *groEL* genes. Transformed cells were grown under kan and chl selection and made electrocompetent. These competent cells were subsequently transformed with plasmid pCW17A1Δ19H encoding the IPTG-inducable CYP17A1 construct, conferring ampicillin (amp)/carbenicillin (carb) resistance, and grown on LB with kan/chlor/carb selection for preparation of glycerol stocks. Recent freezer stocks were used to inoculate 3 mL starter cultures in LB with appropriate antibiotics (kan/chl/carb). These cultures were grown at 37 °C with shaking at 250 rpm until growth was just visible.

Acclimation to M9 minimal media and increasing concentrations of D_2_O was then performed. Standard M9 minimal media containing 1 g L^−1 15^NH_4_Cl (Cambridge Isotope Labs) and trace minerals were used except twice the standard amounts of phosphate salts were added to increase buffering capacity. Advance preparation of the media used the anhydrous form of all salts and filter-sterilized ^15^NH_4_Cl throughout. All media containing D_2_O was filter-sterilized rather than autoclaved to avoid dilution. First, 2 mL of the 3 mL starter culture was inoculated into 100 mL of unlabeled minimal media with kan/chlor/carb supplemented with 50 mg of ^15^N-Isogro (Sigma-Aldrich), and allowed to grow at 37 °C with shaking at 250 rpm until cell density reached 0.6–0.8 at OD_600_ (∼3 h). Second, antibiotics were added to 250 mL of prewarmed 70% D_2_O M9 medium including 0.125 g ^15^N, ^2^H-Isogro, and 2 mL of the previous culture introduced, followed by shaking at 37 °C until cell density reached 0.6–0.8 at OD_600_ (∼6 h). The cells were then pelleted (2740 g for 10 min), gently resuspended, and added to 500 mL of prewarmed 99% D_2_O minimal media, supplemented with 0.25 g of ^15^N, ^2^H-Isogro. This culture was again grown at 37 °C with shaking at 250 rpm until cell density reached 0.6–0.8 at OD_600_ (∼2 h) and were then pelleted as above.

CYP17A1 expression was then accomplished in triply-labeled (^2^H, ^13^C, ^15^N) media. The cell pellet from the previous state was gently resuspended in a few mL of the following media and added to 1 L of 99% D_2_O minimal media, prepared not only with 1 g L^−1 15^NH_4_Cl but also 4 g L^−1 2^H, ^13^C-labeled glucose and 0.5 g ^15^N, ^13^C, ^2^H-Isogro, all in a 2.8 L flask. At this point, 10 μL of 0.5 mg mL^−1^ tetracycline was added to induce chaperone expression. Additionally, 3.5 mg of the CYP17A1 inhibitor abiraterone in 100 μL ethanol was added to achieve a final concentration of 10 μM. Cells continued growing at 37 °C with shaking at 250 rpm until an OD_600_ of 0.8–1.0 was reached (∼1.5 h). At this point, 80 mg of filter-sterilized heme precursor δ-aminolevulinic acid and 240 mg of IPTG were both dissolved in D_2_O and added. Temperature was reduced to 25 °C and shaking speed reduced to 120 rpm for ∼48 hours. Cells were harvest by centrifugation at 4600*g* for 10 min and resuspended in resuspension buffer (50 mM Tris–HCl pH 7.4, 300 mM NaCl and 20% glycerol) before freezing.

Purification through all of the following steps was completed at 4 °C. The thawed cell pellet was supplemented with a pinch of PMSF and abiraterone (in ethanol) to 10 μM. Resuspended cells were homogenized several times using a Dounce homogenizer, rinsing with extra resuspension buffer as needed to collect all cells. This was followed by sonication on ice 8 times for 30 seconds each. The sonicated solution was centrifuged at ∼5000*g* for 10 min at 4 °C. The supernatant was retained and supplemented with 10 μM abiraterone (200 μM in ethanol). CYP17A1 was extracted from the supernatant membranes by adding 2% Emulgen 913 and stirring for one hour. Subsequent centrifugation at 100 000*g* for one hour separated the membrane-containing pellet from the P450-containing supernatant. This supernatant was loaded onto a 15 mL Ni-NTA (Qiagen) column equilibrated with 5 CV of Ni loading buffer (50 mM Tris–HCl pH 7.4, 300 mM NaCl, 20% glycerol, 0.2% Emulgen-913), washed with 4 CV of Ni wash buffer (50 mM Tris–HCl pH 7.4, 300 mM NaCl, 100 mM glycine, 20% glycerol, 0.2% Emulgen-913) and eluted with 3 CV of Ni elution buffer (Ni wash buffer plus 80 mM histidine). The red elution fractions were pooled and diluted 5-fold with CM wash buffer (50 mM Tris–HCl pH 7.4, 100 mM glycine, 20% glycerol) containing 2% Emulgen 913. The diluted sample was loaded onto a 5 ml HiTrap CM FF column equilibrated with 5 CV CM wash buffer including detergent. The column was washed with 20 CV CM wash buffer without detergent, and eluted with 10 CV CM elution buffer (50 mM Tris–HCl, pH 7.4, 100 mM glycine, 500 mM NaCl, 20% glycerol). The red CM elution fractions were pooled, concentrated using Amicon Ulta Centrifugal filters (30 kDa MWCO).

Samples were prepared for NMR by repeated cycles of dilution with 50 mM potassium phosphate pH 6.5, 50 mM NaCl, 10% D_2_O and centrifugal concentration. Sample was then concentrated to ∼0.23 mM, with a final volume of 600 μL, and transferred to a 5 mm thin-walled 8′′ NMR tube. Some precipitate was noted after several hours in the spectrometer, which was removed by centrifugation prior to commencing long acquisition three-dimensional NMR experiments. The remaining sample remained stable through the acquisition and, based on line widths, was monomeric in solution.

### Expression and preparation of uniformly ^2^H, ^15^N-labeled but selective ^13^C-proline-labeled CYP17A1/abiraterone

This sample was prepared as indicated above for the uniformly triple-labeled sample except that the final 1 L of 99% D_2_O minimal media contained ^2^H-glucose instead of ^2^H, ^13^C-glucose, ^15^N, ^2^H-Isogro instead of ^15^N, ^13^C, ^2^H-Isogro, and was supplemented with 0.1 g ^13^C-proline. Expression proceeded for 72 hours after induction. Purification was as indicated for the triple-labeled sample.

### Collection of multidimensional NMR datasets for *u*-^2^H, ^13^C, ^15^N-CYP17A1/abiraterone

Data sets collected for the ^2^H, ^13^C, ^15^N-CYP17A1/abiraterone include ^1^H, ^15^N TROSY-HSQC, HNCA, HN(CO)CA, HNCACB and ^15^N-edited ^1^H-NOESY. Data collected for a perdeuterated sample prepared with uniform ^15^N labeling and selective ^13^C-labeled proline was a two-dimensional HN(CO) experiment, in which ideally only the ^1^H, ^15^N correlations of residues immediately following a proline would be detected. The experiments used for the sequential assignment of backbone ^1^H, ^15^N and ^13^C resonances of CYP17A1/abiraterone were performed at the Landsman Research Facility (Brandeis University) using a Bruker Avance 800 MHz spectrometer operating at 800.13 MHz, 201.20 MHz and 81.086 MHz for ^1^H, ^13^C and ^15^N respectively. All data was collected at 298 K, in order to maximize sample lifetime. All data sets were collected using the TROSY modification for selection of the narrowest line width component of ^1^H, ^15^N correlations.^14^ All data sets were processed using TopSpin (Bruker), and analyzed using Sparky^15^ and CcpNmr AnalysisAssign.^13^

### Modeling of CYP17A1-cyt*b*_5_ complex

The crystallographic structures of CYP17A1 (PDB entry 3RUK) and cytochrome *b*_5_ (PDB 1ICC) were manually juxtaposed using PyMOL^16^ for an initial best fit with published information from mutation^17,18^ and heme modification,^19^ while minimizing the distance between the heme iron atoms of both proteins. This model was then solvated with explicit water in AMBER20, parameterized with forcefield ff19SB, and subjected to 5000 steps of conjugate gradient minimization in order to remove steric interactions and optimize side chain conformations. Two distance restraints were used to establish Arg–Glu interactions, including a 4.5 Å upper limit between OE1 of Glu 43 (cyt*b*_5_) and NH1 of Arg 358 (CYP17A1) and a 6.5 Å upper limit restraint between the CA of Glu 44 (cyt*b*_5_) and NH1 of Arg 347 (CYP17A1), both with force constants of 20 kcal mol^−1^ Å^−1^. A PDB-format file for the minimized complex is available as ESI.‡

## Results

**Fig. 2:**
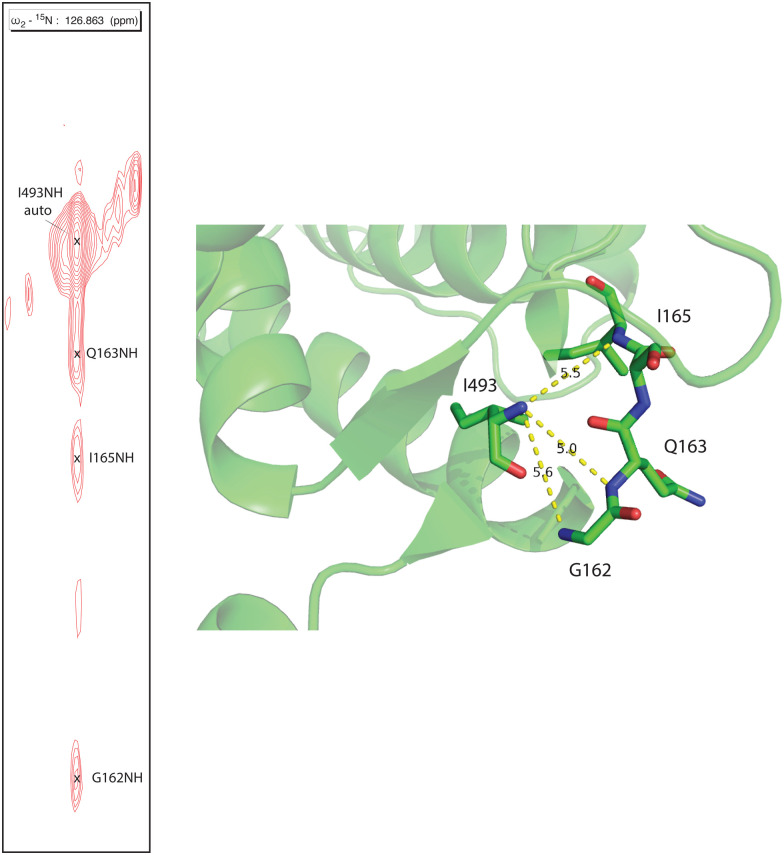
Strip from ^15^N-edited ^1^H, ^1^H NOESY of ^2^H, ^13^C, ^15^N CYP17A1/abiraterone showing NOE connectivity between the amide NH resonance of I493 and those of residues G162, Q163 and I165 in the β5 sheet. Positions of those residues in the 3RUK structure as well as N–N distances are shown at right.

Human CYP17A1 is the first eukaryotic (membrane-bound) cytochrome P450 for which significant sequential backbone ^1^H, ^15^N and ^13^C assignments have been made, and only the fourth including soluble bacterial P450 enzymes. The other enzymes for which assignments are available include the 410-residue CYP101A1 (P450_cam_),^20^ 398-residue MycG^21^ and the 410-residue CYP106A2 (P450_meg_).^22^ In addition to traditional sequential assignment using the through-bond spectra, multiple additional strategies were used to generate these assignments. First, as was the case for the smaller soluble bacterial P450 enzymes, the separate preparation of residue-selective ^15^N labeled samples of the 494-residue membrane CYP17A1 construct was critical for this effort. Identification of backbone ^1^H, ^15^N correlations by residue type provides both multiple starting points for the sequential assignment process as well as confirmation of tentative assignments. Samples selectively ^15^N-labeled with alanine, lysine, arginine, valine, leucine, isoleucine and phenylalanine were prepared for this purpose.^7^ Individual assignments were initially made by mutating a number of individual amino acids of one of these residue types, preparing ^15^N-uniformly labeled samples, and collecting HSQC spectra to identify missing resonances.^7^ A second strategy was employed herein to deal with the 25 proline residues that result in breaks in the amide NH-dependent assignment process. For this purpose, a CYP17A1 sample was prepared uniformly ^15^N labeled combined with specific ^13^C-labeling of only proline. A two-dimensional HN(CO) experiment performed using this sample allowed identification of those residues that are preceded by proline (that is, with a ^13^C(O)–^15^N bond). A third strategy was the collection of an ^15^N-edited NOESY experiment allowing confirmation of assignments by detection of through-space (nuclear Overhauser effects, or NOEs) as opposed to through-bond connectivities. NOEs are typically observed between sequential amide N–^1^H signals in helical and turn structures and across strands in sheets (see Fig. 2).

A complication in the assignment process for CYP17A1 is the fact that the inhibitor abiraterone, which was necessary for stabilizing samples for long-duration acquisitions, ligates the paramagnetic Fe^3+^ (*S* = 1/2) heme, resulting in efficient nuclear spin relaxation within ∼10 Å of the iron. As such, fragmentary connectivity (*e.g.*, NOE combined with selective labeling data) was often needed to make provisional assignments near the heme, including in the B′–C loop, C helix residues 128–131, I helix residues 303–306, K helix and K-β3 loop residues 359–367, β-meander residues 430–446, and β5 extension residues 480–489.

Given the dependence of ^1^H–^15^N–^13^C connectivity on the exchange of ^1^H for ^2^H at NH positions during purification of perdeuterated samples, regions of slow amide exchange account for the remainder of those residues with provisional or missing assignments. These include residues F53 and F54 on the A helix, residue V66–G69 in the β1 sheet, portions of the D and E helices, I helix between residues 312 and 321, and L helix residues between L452 and L460.

**Fig. 3:**
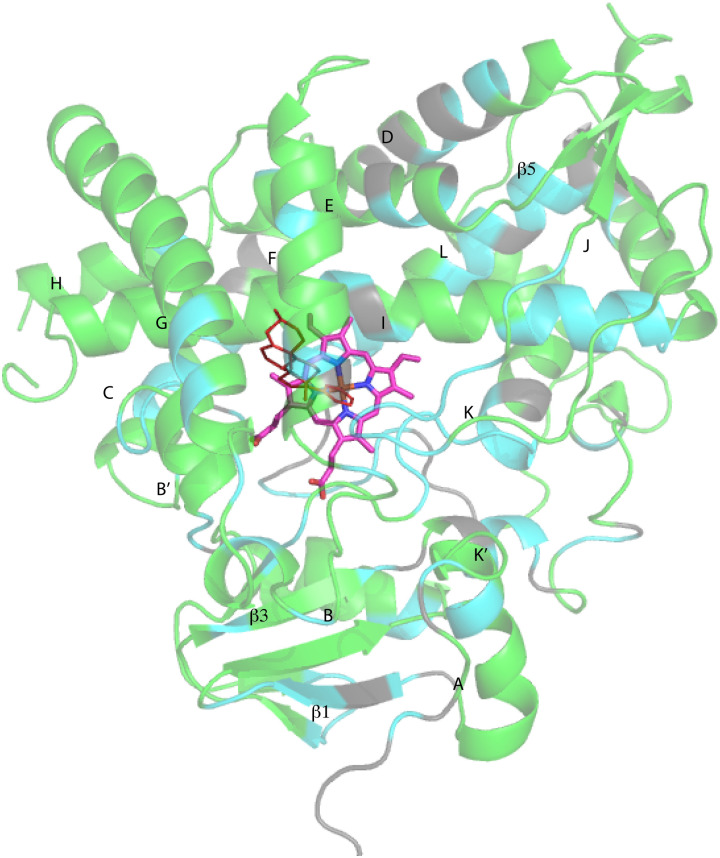
CYP17A1 (PDB entry 3RUK) with abiraterone bound, showing distribution of sequential backbone resonance assignments. Assignments for regions in green are supported by multiple forms of data (see text for details), regions in blue are provisionally assigned based on fewer data. Those shown in grey are not assigned. Secondary structures were assigned by DSSP analysis of PDB 3RUK (see ESI‡) are labeled as first described by Poulos.^23^ Helices: A, M49–Y60; B, H80–L86; B′, A100–A105; C, A119–A133; D, L142–T159; E, S168–F184; F, E194–L209; G′, W120–I123; G, T228–K253; H, M262–N272; I, D289–H320; J, P322–N335; K, L351–L363; K′, L396–H401; K′′, P414–F417; L, E445–R462. Strands: β1, I63–M68, K71–V76, E391–I394, H373–K374; β2, S379–I381, F384–386; β3, F463–E466, K492–V495; β4, I479–P480, F484–L385; β5, F463–Q466, V491–495. Abiraterone is shown in thin red lines, heme is shown in heavier pink lines.

Despite these issues, it was possible to assign backbone resonances for 93% of the non-proline residues in the polypeptide. Of those assignments, 82% are supported by more than one type of data (selective labeling, NOE and through bond connectivity). The distribution of assignments shown in Fig. 3 provides confidence that no significant portion of the enzyme is unrepresented by at least provisional assignments. A tabulation of assignments is provided in ESI.‡

### Conformational heterogeneity in CYP17A1/abiraterone

**Fig. 4:**
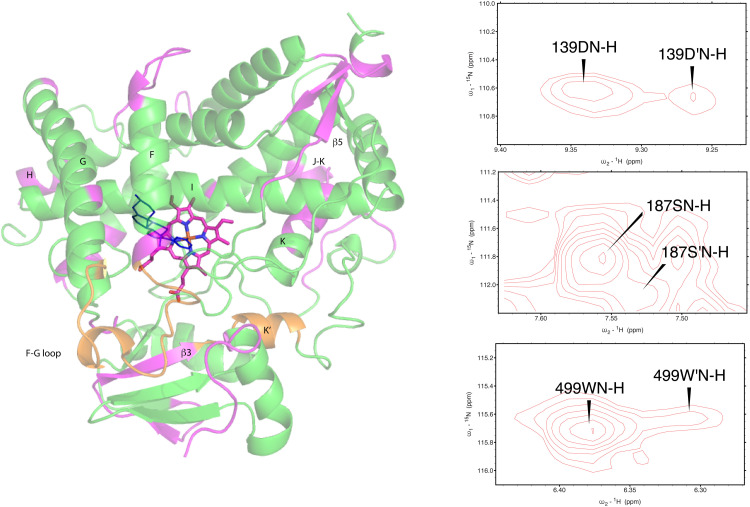
(left) Distribution of doubled resonances (in purple) indicating slow exchange of two conformational ensembles mapped on the CYP17A1/abiraterone X-ray structure (3RUK). The F–G loop and K′ helix shown in orange show intermediate exchange, as evidenced by significant broadening of resonances. (right) Examples of doubling of correlations in ^1^H, ^15^N-TROSY HSQC spectrum of CYP17A1/abiraterone, Asp 139 (C–D loop), Ser 187 (E–F loop) and Trp 499 (β5 strand).

**Fig. 5:**
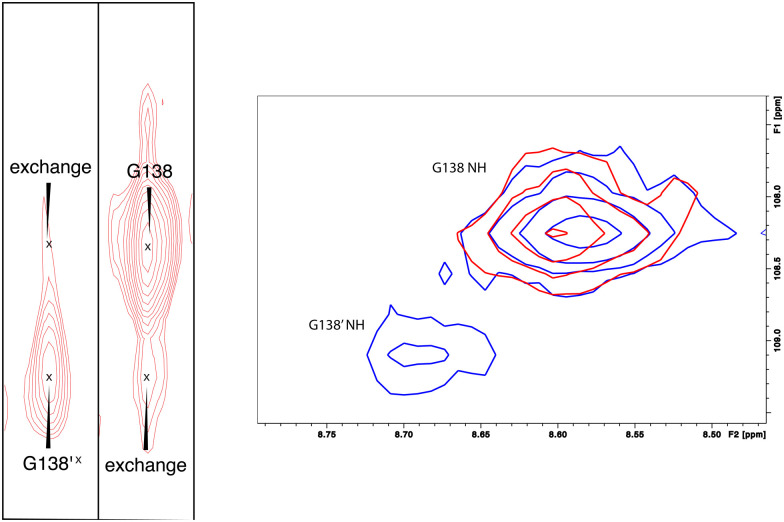
(left) Conformational exchange detected in ^15^N-edited NOESY of CYP17A1/abiraterone for Gly 138 (C–D loop). Autocorrelation peaks for G138 and G138′ are indicated, as are exchange cross peaks. (right) Collapse of states observed in CYP17A1/abiraterone ^1^H, ^15^N-TROSY HSQC for Gly 138 in the absence of cyt*b*_5_ (blue) to a single conformation (red) upon addition of 0.4 equivalents of cyt*b*_5_.

As was reported previously, evidence for two conformational ensembles at slow exchange on the ^1^H chemical shift time scale was detected in multiple regions of the CYP17A1/abiraterone structure.^7^ Several of the best-resolved examples are shown in Fig. 4, as is the structural distribution of observed doubling. Although the examples mentioned and highlighted in Fig. 4 are among the clearest examples of discrete conformations, many other residues show significant distortion in peak shape. Where resolved, the split correlations exhibit volume ratios of approximately 7 : 1. The rate of exchange between the two forms can be calculated from the ratio of exchange cross peak volumes to the autocorrelation diagonal peak in ^15^N-edited NOESY data for the well-resolved case of Gly 138 in the C–D loop (see Fig. 5). Taking into account the NOESY mixing time (100 ms), the conformational exchange in the C–D loop region is ∼300 s^−1^. As described in the next section, many of these correlations show evidence of collapse to a single conformation upon addition of cyt*b*_5_.

The situation in the F–G loop (L209–T228) that caps the active site is more complex. In this region, “smearing” of correlations, rather than a discrete two state occupancy, suggest that conformational exchange is intermediate (rather than slow, as implied by discrete correlations) on the ^1^H chemical shift time scale. Similarly, residues in the K′ helix, which we have found to be sensitive to substrate binding and orientation in CYP101A1^24^ and MycG,^25^ are also “smeared”. We note that some crystallographic structures of CYP17A1 have a second lower-occupancy ligand binding site near the F–G loop that affects conformations in this region, so smearing may be the result of abiraterone exchanging at an intermediate rate in the second site.^26,27^

Also seen was doubling in the loop region between the H and I helices, which is disordered in the 3RUK structure. Indeed, the relatively narrow linewidths observed for this loop indicate that this region is mobile in solution. While the ratio of the two conformations based on peak integrations is ∼7 : 1, these residues do not collapse to a single peak upon addition of cyt*b*_5_.

### Perturbations upon cyt*b*_5_ binding to CYP17A1/abiraterone

**Fig. 6:**
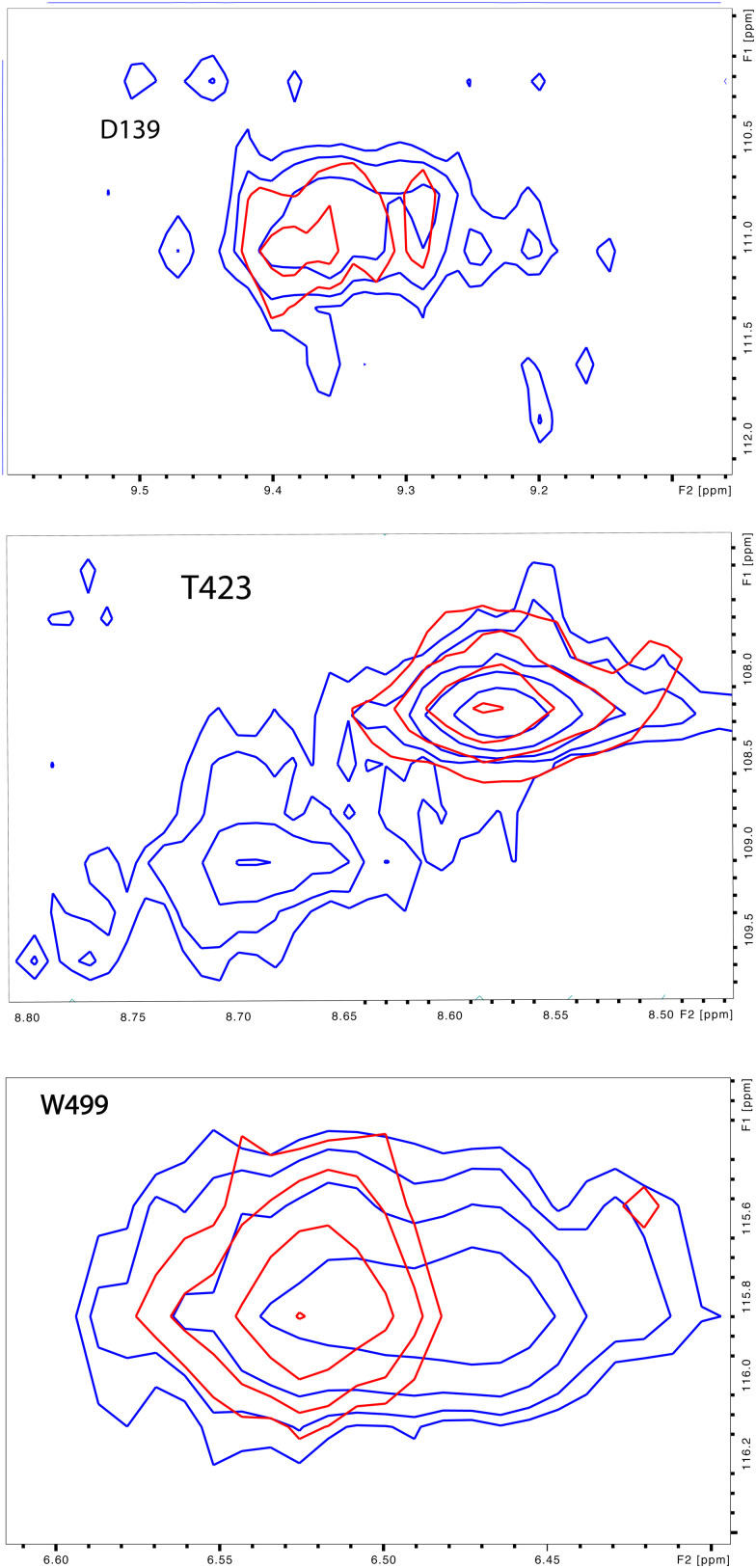
Preferential population of CYP17A1/abiraterone major conformation upon binding of cyt*b*_5_. ^1^H–^15^N correlations for Asp 139 (C–D loop), Thr 423 (β-meander) and Trp 499 (β5 strand) in the absence (blue) and presence (red) of 0.4 equivalents of cyt*b*_5_.

**Fig. 7:**
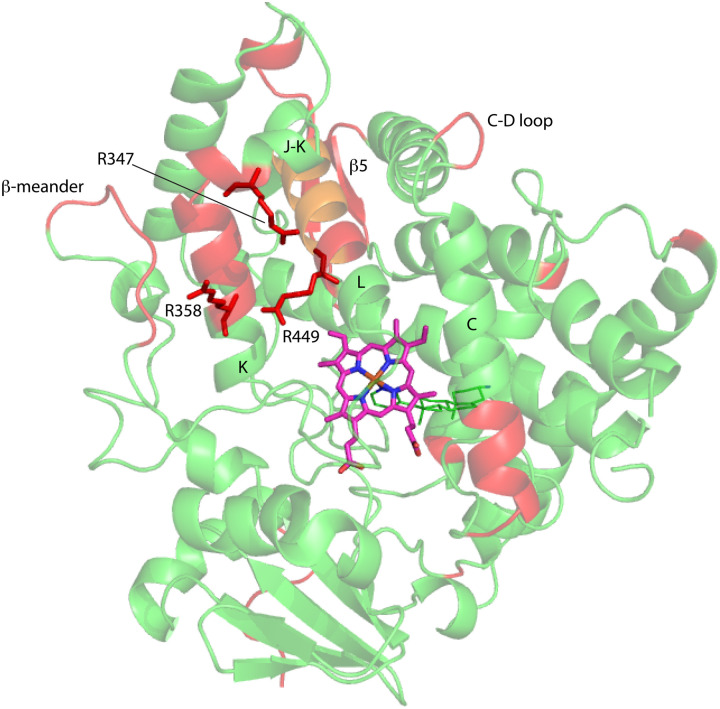
Distribution of regions (in red) showing conformational selection in CYP17A1/abiraterone upon binding of cyt*b*_5_. The view is of the proximal surface, rotated ∼60° counterclockwise from Fig. 3. Regions shown in orange are tentative, either due to spectral overlap or provisional resonance assignment. The side chains of arginine residues (R347, R358 and R449) implicated in cyt*b*_5_ binding are shown in red.

Upon comparison of ^1^H, ^15^N-TROSY HSQC experiments performed on CYP17A1/abiraterone in the absence and presence of 0.4 equivalents of cyt*b*_5_, it was found that many of those doubled or broadened correlations are driven to primarily populate the more abundant conformational ensemble. An example of this is shown in Fig. 5 for Gly 138 in the C–D loop, while several other resolved examples are shown in Fig. 6. Given the spectral crowding in many regions of the HSQC, it was not possible to confirm that such collapse happens in all cases of doubling or broadening that were identified. However, sufficient examples were found to indicate that the collapse occurs in regions adjacent to the proposed cyt*b*_5_ binding site on the proximal face of CYP17A1, as well as in several regions on the distal (active site) side of the heme, including portions of the B–B′ loop, the C-terminal end of helix F, the N-terminal end of helix G as well as the β5 sheet and C-terminal helix following the β5 sheet. This indicates the existence of mechanical coupling pathways between the cyt*b*_5_ binding site and regions of the enzyme involved in substrate binding and orientation (Fig. 7).

**Fig. 8:**
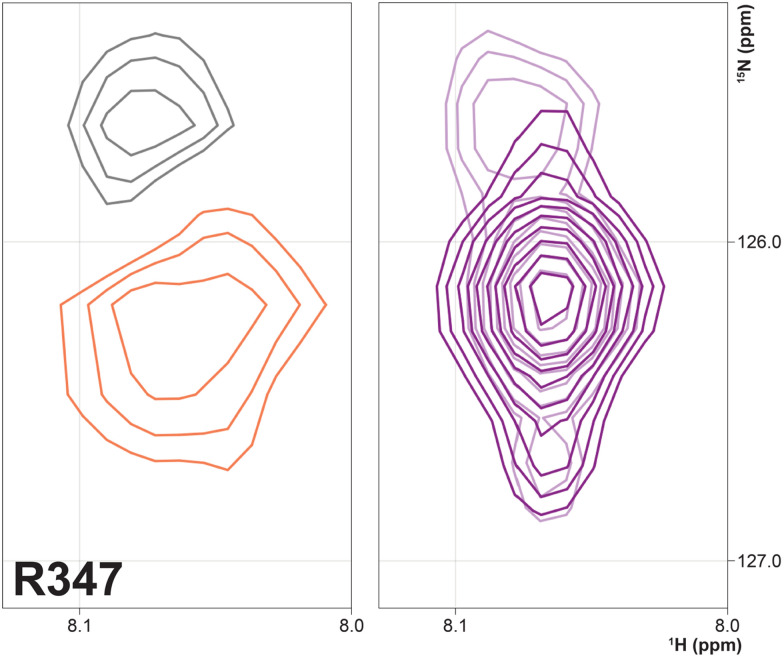
(right) Overlay of ^1^H–^15^N correlation for Arg 347 in the absence (light contours) and presence (dark contours) of 0.4 equivalents of cyt*b*_5_. (left) Spectral subtraction of the same correlation with black contours representing loss of signal and orange showing increased intensity upon addition of cyt*b*_5_.

Three arginine residues on the proximal side of CYP17A1 have been implicated in the interaction with cyt*b*_5_, in that their mutation prevents the lyase reaction but does not interfere with 17-hydroxylase activity.^18^ These solvent-exposed arginines—Arg 347 (on a short helical segment between the J and K helices), Arg 358 (K helix) and Arg 449 (L helix)—are likely to participate in forming the cyt*b*_5_–CYP17A1 complex as detected by NMR.^9^ Backbone correlations for all three residues have now been assigned, as have many other residues in the vicinity of the proposed cyt*b*_*5*_ binding site. The correlation for Arg 347, while not obviously doubled, is broadened asymmetrically and collapses to a single peak upon addition of cyt*b*_5_ (Fig. 8).

Most resonances assigned in this region show evidence for the ∼7 : 1 doubling described above (Fig. 4), including an exchangeable ^1^H signal not coupled to ^15^N detected ∼ 4 ppm (^1^H), with strong NOE connectivity to a threonine residue, Thr 356 on the K helix, and so is assigned as the Thr 356 γ-OH. As hydroxyl protons typically exchange too rapidly to be detected separately from bulk water, the Thr 356 γ-OH proton is protected from fast exchange either by isolation from bulk solvent and/or strong hydrogen bonding. The nearest neighbors of the γ-OH of Thr 356, the side chains of Val 324 and Leu 328 (J helix), Leu 352 and Leu 353 (K helix), Phe 412 and Pro 414 (both part of the β-meander), are all hydrophobic. With no obvious hydrogen bonding partners present, the slow exchange indicates that solvent accessibility is limited between the J and K helices as well as the β-meander leading to the axial cysteine thiolate heme ligand, Cys 442. This observation further indicates that any motion or displacement involving these secondary structural features is likely to be tightly coupled.

The ratio of the integrated peak volumes between the two conformations, as well as the relatively slow rate of exchange between them, suggests a thermodynamically controlled X-Pro *cis*/*trans* isomerization, with the binding of cyt*b*_5_ driving population of the thermodynamically-favored conformer. We have previously observed such selection in a proline-rich loop in human acireductone dioxygenase upon binding of a regulatory peptide.^28^ Pro 342 in the J–K loop and Pro 420 in the β-meander are both in regions affected by cyt*b*_5_ binding. Neither are located in tight turns or other regular secondary structures, and so could have sufficient conformational freedom to adopt either conformation. The preceding residue in each case (Thr 341 and Asn 419) could provide hydrogen bonding options for improved stabilization of the *cis* conformer, which brings the proline carbonyl close to the side chain of the preceding residue. Given the proximity of the J–K loop to many of the affected residues shown in Fig. 7, Pro 342 would seem the more likely candidate if such an isomerization is indeed responsible for the observed conformational heterogeneity. In support of this hypothesis, in the HN(CO) experiment identifying residues i + 1 to proline, the NH correlation of Thr 343 shows significant splitting, while Ala 421 does not. However, there is at present insufficient data to confirm that either proline is in fact responsible for the observed heterogeneity.

## Discussion

Previous work has shown that, at least in the case of the most well-characterized P450, the camphor hydroxylase cytochrome P450_cam_ (CYP101A1), regulation of the second electron transfer required for turnover occurs through the interaction of CYP101A1 with its physiological redox partner and effector, the Fe_2_S_2_ ferredoxin putidaredoxin.^29–32^

The current case differs from the bacterial CYP101A1 system in several regards. The most significant is that there are two proteins (NADPH-cytochrome P450 reductase (CPR) and cyt*b*_5_) that can interact directly with CYP17A1, with evidence that the binding sites of CPR and cyt*b*_5_ at least partially overlap and that their binding to CYP17A1 is competitive.^10^ Parsing the roles of each partner in these complexes is therefore more difficult. Furthermore, *in vivo*, all three proteins (cyt*b*_5_, CYP17A1 and CPR) are membrane-bound: both cyt*b*_5_ and CYP17A1 used in the current experiments have been modified to remove their terminal transmembrane helices. Still, it is expected that membrane binding effectively limits the surfaces available for interaction between the proteins, reducing the dimensionality of the problem. Evidence that the full-length and truncated CYP17A1 enzymes act similarly are that they bind the same substrates and inhibitors, and produce the same hydroxylase and lyase products.^33^

The role of cyt*b*_5_ in stimulating the lyase reaction likely involves both reduction of the oxygen-heme-substrate complex, as well as allosteric conformational shifts. While redox-inactive manganese-heme reconstituted cyt*b*_*5*_ does not increase the rate of the lyase reaction,^34^ Fe-cyt*b*_5_ was found to stimulate lyase activity, and reduces oxy-ferrous CYP17A1 ∼10× faster than CPR.^35^ Recent evidence obtained using nanodisc-bound cyt*b*_5_ and CYP17A1 with intact membrane-binding domains supports a mechanism for the lyase reaction involving a peroxo intermediate leading to an electrocyclic cleavage of the C17–C20 bond, rather than the compound I-mediated hydrogen abstraction responsible for the C17 hydroxylation.^36^ Based on substrate-dependent changes in Raman-active Fe–O vibrational frequencies, those workers also propose that a hydrogen bond formed between the 17-OH of 17-hydroxyprogesterone and the Fe-bound peroxo species switches from the terminal oxygen to the Fe-bound oxygen atom upon cyt*b*_5_ binding. It has been shown that rate enhancement for the lyase reaction by cyt*b*_5_ is greater for 17-hydroxyprogesterone (∼20-fold) than for 17-hydroxypregnenalone (∼8-fold).^37^

Our current results, especially the conformational selection occurring in regions adjacent to the active site (the β5 sheet and F and G helices) may rationalize the differences in cyt*b*_5_'s stimulation of lyase activity for the two substrates. The C3 hydroxyl group of pregnenolone and C3 carbonyl of progesterone are observed in crystallographic structures to interact with Asn 202 on the F helix, and Val 482 and 483 on the loop between the β5 strands lie adjacent to the steroid skeleton. Either or both interactions could provide mechanical coupling pathways to transmit cyt*b*_5_-induced conformational selection to reposition 17-hydroxyprogesterone more appropriately for the lyase reaction than 17-hydroxypregnenolone.

**Fig. 9:**
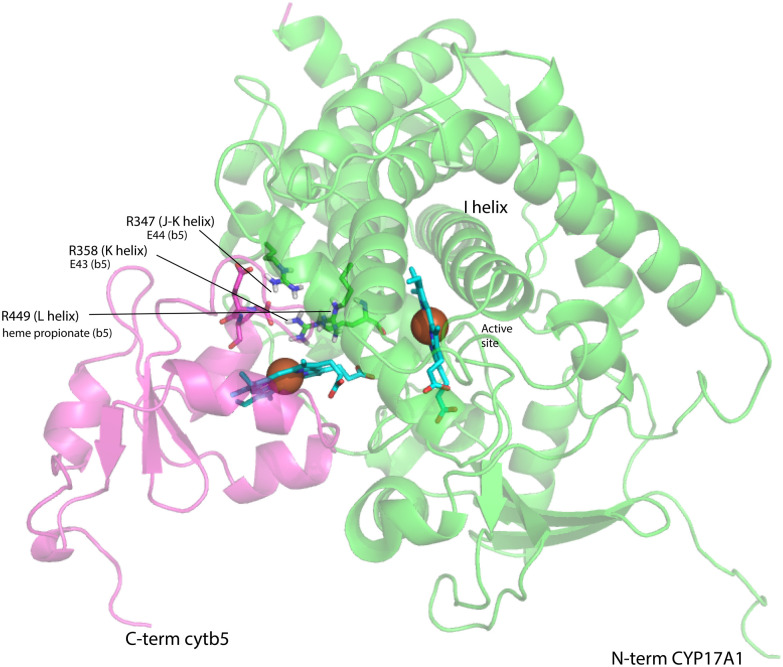
Model for a complex formed by cyt*b*_5_ on the proximal face of CYP17A1, placing Arg 347, Arg 358 and Arg 449 adjacent to Glu 44, Glu 43 and a cyt*b*_5_ heme propionate, respectively. Both heme iron atoms are shown as orange spheres. CYP17A1 is rotated counterclockwise ∼90° and tilted clockwise ∼20° from the orientation in Fig. 3 membrane binding domains of both proteins are not shown, but are extensions of the C-term of cyt*b*_5_ and N-term of CYP17A1 that are oriented toward the heavy black line at the bottom of the figure. Crystal structures 1ICC and 3RUK were used as templates. Model is courtesy of Prof. Eliana Asciutto, UNSAM, Buenos Aires, AG.

On the other hand, our results are limited to those regions of CYP17A1 that are accessible to solution NMR, that is, those regions far enough from the heme iron to not undergo significant paramagnetically-induced relaxation. It is clear is that binding of cyt*b*_5_ to CYP17A1/abiraterone does not lead to large conformational changes in the regions accessible to NMR, but rather drives the population of an already favored conformer. These changes seem unlikely to explain the considerable overall acceleration of lyase activity by cyt*b*_5_. However, given that cyt*b*_5_-induced perturbations are observed in the β-meander containing the the axial cysteine thiolate ligand, we speculate that more profound changes may take place at the proximal face of the heme upon cyt*b*_5_ binding, transmitted *via* concerted motions of the J–K, K and L helices and the β-meander implied by the slow exchange of the Thr 356 γ-OH proton described above. Such changes could include modulation of heme vibrational modes and/or modified iron position relative to the equatorial pyrrole nitrogen ligands transmitted through the axial Fe–S bond. The enhancement of lyase activity by cyt*b*_5_ may be attributable to such changes, especially if cyt*b*_5_ is a better “fit” than CPR at the site defined by Arg 347, Arg 358 and Arg 449, the positive charges on which are required for the lyase reaction.^18^ A plausible model for the cyt*b*_5_-CYP17A1 complex (Fig. 9) orients the membrane-binding domains of both proteins (the C-terminal of cyt*b*_5_ and N-terminal of CYP17A1) in the same direction. As electron transfer likely takes place in the complex, the heme irons of both cyt*b*_5_ and CYP17A1 are in close proximity, and the three arginine residues on CYP17A1 interact with negative charges on cyt*b*_5_: Glu 43 (cyt*b*_5_) with Arg 347, Glu 44 (cyt*b*_5_) with Arg 358 and a cyt*b*_5_ heme propionate with Arg 449. This juxtaposition places the nearest porphyrin γ-carbon of cyt*b*_5_ at 11.5 Å from the heme iron of CYP17A1, with a 15.0 Å distance between the heme irons.

The research described here confirms the importance of NMR for characterizing the response of CYP17A1 structure to perturbations induced by cyt*b*_5_ binding. To overcome the problem of paramagnetic relaxation and extend the current assignments into regions closer to the heme, a diamagnetic ferrous (Fe^2+^) form of the heme must be stabilized. We have recently described a pregnanolone-derived compound, 3-formyl-(*R*)-pregnane-20-isonitrile, that is capable of forming a dative Fe^2+^–C bond, and accomplishes this stabilization.^27^ We expect this diamagnetic form of CYP17A1 will allow us to assign many of the resonances currently inaccessible to multidimensional NMR methods.

## Author contributions

Alaina M. Richard: data curation; formal analysis; writing – review & editing. D. Fernando Estrada: investigation (generation, data collection, and analysis of CYP17A1 with specific amino acid labeling (WT and mutants); generation and data collection of all CYP17A1 samples +/− cyt*b*_5_); formal analysis; writing – review & editing. Liam Flynn: investigation (^15^N-Lys sample preparation & data acquisition). Susan Sondej Pochapsky: investigation (data collection for triple-labeled CYP17A1 and ^13^C-Pro CYP17A1 and ^15^N-Lys sample). Emily E. Scott: conceptualization; funding acquisition; investigation (generation and data collection of triple-labeled CYP17A1 and ^13^C-Pro CYP17A1); project administration; supervision; writing – review & editing. Thomas C. Pochapsky: formal analysis; funding acquisition; methodology; project administration; supervision; validation; visualization; writing – original draft; writing – review & editing.

## Conflicts of interest

There are no conflicts to declare.

## Supplementary Material

CP-026-D4CP01268B-s001

CP-026-D4CP01268B-s002

CP-026-D4CP01268B-s003
