# Supplementary material for: Tracking protein–protein interactions by NMR: conformational selection in human steroidogenic cytochrome P450 CYP17A1 induced by cytochrome b5
Source: Phys Chem Chem Phys. 2024 May 31;26(24):16980–8. doi: 10.1039/d4cp01268b (PMC11186455; doi:10.1039/d4cp01268b)
Supplement: CP-026-D4CP01268B-s003 [file CP-026-D4CP01268B-s003.pdf]

Electronic Supplementary Material (ESI) for Physical Chemistry Chemical Physics.
This journal is © the Owner Societies 2024

Sequence Alignment and Secondary Structure (DSSP)

```
2A6:  secondary structure from 1Z10 (coumarin complex)
2A13: secondary structure from Scott lab structure
2B4:  secondary structure from 1SUO (4PI complex)
2E1:  secondary structure from ???
2A17: secondary structure from abiraterone structure for mol A/B, C/D, consensus

           1                                                    50
     2A6   MLASGMLLVA LLVCLTVMVL MSVWQQRKSK G.KLPPGPTP LPFIGNYLQL
    2A13   MLASGLLLVT LLACLTVMVL MSVWRQRKSR G.KLPPGPTP LPFIGNYLQL
     2B4   MEFSLLLLLA FLAGLLLLLF RG...HPKAH G.RLPPGPSP LPVLGNLLQM
     2E1   MSALGVT.VA LLVWAAFLLL VSMWRQVHSS W.NLPPGPFP LPIIGNLFQL
    17A1   M....WELVA LLLLTLAYLF ...WPKRRCP GAKYPKSLLS LPLVGSLPFL 43
 17A1Δ19   M....~~~~~ ~~~~~~~~~~ ~~~~~~AKKT GAKYPKSLLS LPLVGSLPFL
                                                             A'

           51                                                  100
     2A6   NTE.QMYNSL MKISERYGPV FTIHLGPRRV VVLCGHDAVREA LVDQAEEF
    2A13   NTE.QMYNSL MKISERYGPV FTIHLGPRRV VVLCGHDAVKEA LVDQAEEF
     2B4   DRK.GLLRSF LRLREKYGDV FTVYLGSRPV VVLCGTDAIREA LVDQAEAF
     2E1   ELK.NIPKSF TRLAQRFGPV FTLYVGSQRM VVMHGYKAVKEA LLDYKDEF
  AB17A1   PRHGHMHNNF FKLQKKYGPI YSVRMGTKTT VIVGHHQLA EV LIKKGKDF 93
  CD17A1   PRHGHMHNNF FKLQKKYGPI YSVRMGTKTT VIVGHHQLA EV LIKKGKDF 93
    17A1   PRHGHMHNNF FKLQKKYGPI YSVRMGTKTT VIVGHHQLA EV LIKKGKDF 93
                   A            β1-1      β1-2         B

           101                                                 150
     2A6   SGRGEQATFD WVF.KGYGVV FS.NGERAKQ LRRFSIATLR DFGVGKRGIE
    2A13   SGRGEQATFD WLF.KGYGVA FS.NGERAKQ LRRFSIATLR GFGVGKRGIE
     2B4   SGRGKIAVVD PIF.QGYGVI FA.NGERWRA LRRFSLATMR DFGMGKRSVE
     2E1   SGRGDLPAFH .AH.RDRGII FN.NGPTWKD IRRFSLTTLR NYGMGKQGNE
  AB17A1   SGRPQMATLD IASNNRKGIA FADSGAHWQL HRRLAMATFA LFKDGDQKLE 143
  CD17A1   SGRPQMATLD IASNNRKGIA FADSGAHWQL HRRLAMATFA LFKDGDQKLE 143
    17A1   SGRPQMATLD IASNNRKGIA FADSGAHWQL HRRLAMATFA LFKDGDQKLE 143
            β1-5     B'                      C                  D

           151                                                 200
     2A6   ERIQEEAGFL IDALRGTGGA NIDPTFFLSR TVSNVISSIV FGDRFDYKDK
    2A13   ERIQEEAGFL IDALRGTHGA NIDPTFFLSR TVSNVISSIV FGDRFDYEDK
     2B4   ERIQEEARCL VEELRKSKGA LLDNTLLFHS ITSNIICSIV FGKRFDYKDP
     2E1   SRIQREAHFL LEALRKTQGQ PFDPTFLIGC APCNVIADIL FRKHFDYNDE
  AB17A1   KIICQEISTL CDMLATHNGQ SIDISFPVFV AVTNVISLIC FNTSYKNGDP 193
  CD17A1   KIICQEISTL CDMLATHNGQ SIDISFPVFV AVTNVISLIC FNTSYKNGDP 193
    17A1   KIICQEISTL CDMLATHNGQ SIDISFPVFV AVTNVISLIC FNTSYKNGDP 193
                D                           E
```

```
        201                                               250
2A6     EFLSLLRMML GIFQFTSTST GQLYEMFSSV MKHLPGPQQQ AFQLLQGLE
2A13    EFLSLLRMML GSFQFTATST GQLYEMFSSV MKHLPGPQQQ AFKELQGLE
2B4     VFLRLLDLFF QSFSLISSFS SQVFELFSGF LKHFPGTHRQ IYRNLQEIN
2E1     KFLRLMYLFN ENFHLLSTPW LQLYNNFPSF LHYLPGSHRK VIKNVAEVK
AB17A1  ELNVIQNYNE GIIDNLSKDS LVDLVPWLKI FPN..KTLEK LKSHVKIRN  240
CD17A1  ELNVIQNYNE GIIDNLSKDS LVDLVPWLKI FPN..KTLEK LKSHVKIRN  240
17A1    ELNVIQNYNE GIIDNLSKDS LVDLVPWLKI FPN..KTLEK LKSHVKIRN  240
                   F          F'      G'                 G

251                                             300
2A6     DFIAKKVEHN QRTLDPNSPR DFIDSFLIRM QEEEKNP... ...NTEFYLK
2A13    DFIAKKVEHN QRTLDPNSPR DFIDSFLIRM QEEEKNP... ...NTEFYLK
2B4     TFIGQSVEKH RATLDPSNPR DFIDVYLLRM EKDKSDP... ...SSEFHHQ
2E1     EYVSERVKEH HQSLDPNCPR DLTDCLLVEM EKEKHSA... ...ERLYTMD
AB17A1  DLLNKILENY KEKFRSDSIT NMLDTLMQAK MNSDNGNAGP DQDSELLSDN  290
CD17A1  DLLNKILENY KEKFRSDSIT NMLDTLMQAK MNSDNGNAGP DQDSELLSDN  290
17A1    DLLNKILENY KEKFRSDSIT NMLDTLMQAK MNSDNGNAGP DQDSELLSDN  290
                                       H

        301                                         350
2A6     NLVMTTLNLF IGGTETVSTT LRYGFLLLMK HPEVEAKVHE EIDRVIGKNR
2A13    NLVMTTLNLF FAGTETVSTT LRYGFLLLMK HPEVEAKVHE EIDRVIGKNR
2B4     NLILTVLSLF FAGTETTSTT LRYGFLLMLK YPHVTERVQK EIEQVIGSHR
2E1     GITVTVADLF FAGTETTSTT LRYGLLILMK YPEIEEKLHE EIDRVIGPSR
AB17A1  HILTTIGDIF GAGVETTTSV VKWTLAFLLH NPQVKKKLYE EIDQNVGFSR  340
CD17A1  HILTTIGDIF GAGVETTTSV VKWTLAFLLH NPQVKKKLYE EIDQNVGFSR  340
17A1    HILTTIGDIF GAGVETTTSV VKWTLAFLLH NPQVKKKLYE EIDQNVGFSR  340
                          I                          J

        351                                         400
2A6     QPKFEDRAKM PYMEAVIHEI QRFGDVIPMS LARRVKKDTK FRDFFLPKGT
2A13    QPKFEDRAKM PYTEAVIHEI QRFGDMLPMG LAHRVNKDTK FRDFFLPKGT
2B4     PPALDDRAKM PYTDAVIHEI QRLGDLIPFG VPHTVTKDTQ FRGYVIPKNT
2E1     IPAIKDRQEM PYMDAVVHEI QRFITLVPSN LPHEATRDTI FRGYLIPKGT
AB17A1  TPTISDRNRL LLLEATIREV LRLRPVAPML IPHKANVDSS IGEFAVDKGT  390
CD17A1  TPTISDRNRL LLLEATIREV LRLRPVAPML IPHKANVDSS IGEFAVDKGT  390
17A1    TPTISDRNRL LLLEATIREV LRLRPVAPML IPHKANVDSS IGEFAVDKGT  390
           J'            K                  β1-4   β2-1  β2-2
```

```
        401                                                 450
   2A6  EVYPMLGSVL RDPSFFSNPQ DFNPQHFLNE KGQ..FKKSD AFVPFSIGKR
  2A13  EVFPMLGSVL RDPRFFSNPR DFNPQHFLDK KGQ..FKKSD AFVPFSIGKR
   2B4  EVFPVLSSAL HDPRYFETPN TFNPGHFLDA NGA..LKRNE GFMPFSLGKR
   2E1  VVVPTLDSVL YDNQEFPDPE KFKPEHFLNE NGK..FKYSD YFKPFSTGKR
AB17A1  EVIINLWALH HNEKEWHQPD QFMPERFLNP AGTQLISPSV SYLPFGAGPR 440
CD17A1  EVIINLWALH HNEKEWHQPD QFMPERFLNP AGTQLISPSV SYLPFGAGPR 440
  17A1  EVIINLWALH HNEKEWHQPD QFMPERFLNP AGTQLISPSV SYLPFGAGPR 440
         β1-3     K'                K″                         K'''

        451                                                 500
   2A6  NCFGEGLARM ELFLFFTTVM QNFRLKSSQS PKDIDVSPKH VGFATIPRNY
  2A13  YCFGEGLARM ELFLFFTTIM QNFRFKSPQS PKDIDVSPKH VGFATIPRNY
   2B4  ICLGEGIART ELFLFFTTIL QNFSIASPVP PEDIDLTPRE SGVGNVPPSY
   2E1  VCAGEGLARM ELFLLLCAIL QHFNLKPLVD PKDIDLSPIH IGFGCIPPRY
AB17A1  SCIGEILARQ ELFLIMAWLL QRFDLEVPDD GQLPSLEG.I PKVVFLIDSF 489
CD17A1  SCIGEILARQ ELFLIMAWLL QRFDLEVPDD GQLPSLEG.I PKVVFLIDSF 489
  17A1  SCIGEILARQ ELFLIMAWLL QRFDLEVPDD GQLPSLEG.I PKVVFLIDSF 489
                     L           β3-3          L'       β4-1  β4-2

   2A6  TMSFLPR 494
  2A13  TMSFLPR 494
   2B4  QIRFLAR
   2E1  KLCVIPRS
AB17A1  KVKIKVRQAW REAQAEGST 508
CD17A1  KVKIKVRQAW REAQAEGST 508
CD17A1  KVKIKVRQAW REAQAEGST 508
         β3-2
```
